# Annotations

**Published:** 1906-06-16

**Authors:** 


### ANNOTATIONS.

### "Improving" London.

The word "improving" is used by a daily paper in a sarcastic way when commenting upon the changes which have taken place in recent years in the centre and in the outskirts of London. There can scarcely be two opinions as to whether, from the architectural standpoint, the most important streets of London have altered for the better; but many "improvements" have destroyed the dwellings of poor inhabitants and driven them to seek homes in or near the periphery of the Metropolis. The need for accommodation has been met by the speculative builder, who has transformed what were garden suburbs into closely packed and unsightly collections of small houses. This, unfortunately, is not only true of houses built for artisans, but of those intended for classes higher in the social scale. Wherever the speculative builder acquires land his main object seems to be to crowd as much bricks and mortar as possible upon a given space. This is a free country, but freedom too often permits some member of the community, for personal gain, to rob us of much of what of all things should be obtained in as large a measure as possible by everyone of us—fresh air and sunlight. In one sense the builder is not to blame. While people are willing to live in houses placed so close to one another as to allow those who dwell in them to know almost as much about their neighbours' affairs as their own, houses thus situated will continue to be built. That so many should be content with the deprivation of light, air, and privacy that this close packing entails is mysterious. Where circumstances compel residence in some of the busier parts of London, natural instincts and inclinations may have to be subordinated to necessity, but a daily journey to and fro from the suburbs should be compensated by a larger measure of those blessings which Nature can only fully bestow away from the crowded areas.

### High Frequency Currents and Surface Temperatures.

The associations of electricity in the history of medical practice have not been altogether fortunate ones. Electrical methods have suffered from the enthusiasm of injudicious friends and from adoption by those having only imperfect training and experience. Worse still, they have not infrequently fallen into doubtful company. Hence, it is not altogether surprising that many practitioners banish electricity from their list of curative agencies and receive with an incredulous smile the statements of those who cultivate such forms of treatment. What is true in this respect of electrical methods generally has been illustrated in recent times in the case of that particular form known as high frequency currents. The therapeutic position of these currents will, of course, be gradually determined as a result of experience, and in the meantime this must be allowed to accumulate. But that they have no effect whatever on bodily function is clearly disproved by a series of observations recently submitted to the British Electro-therapeutic Society by Dr. W. F. Somerville. In these it is shown that the passage of high frequency currents through the body produces a rise, often a very considerable rise, of the surface temperature. The movement of the mercury was found in many cases to reach, or even to exceed, 10° F. It is suggested that such a result is due to dilatation of the blood-vessels, as a consequence of the action of the electrical currents of the vasomotor apparatus, and that, in the same way, many of the curative values claimed for this method may be explained. Whether this is so or not, it cannot be denied that Dr. Somerville's contribution is a sound piece of scientific work, and that it establishes a presumptive claim for attention to high frequency currents even on the part of those who have hitherto been most sceptical of the various therapeutic results attributed to this particular electrical method.

### What is Butter?

The need for some fixed standard for butter was shown in a case recently dealt with at Skipton, where a farmer was summoned for selling as butter a substance which the county analyst for the West Riding of Yorkshire declared to contain 33 per cent. of margarine. This gentleman had founded his opinion on the absence in the butter submitted to him of a proper proportion of those volatile fatty acids which differentiate true butter from that adulterated with fat from the carcase of an animal. But against this opinion was set that of Mr. Otto Ernhardt, public analyst for Nottinghamshire, who declared that it was impossible to fix a chemical standard for butter fat. The milk from which the butter was made was abnormally low in quality, and hence the butter made from it was so inferior. The farmer was exonerated from the charge of adulteration, rightly, no doubt; but it is easy to see that the publication of such a case may make it easy for those who adulterate their butter to do so with impunity. The method of testing butter generally employed is what is called the Wolny method. A solution of alkali of a particular standard is applied to the butter under investigation, and if under this solution the butter shows a proportion of volatile fatty acids represented by the figure 28, it is assumed that the butter is pure. If less than that figure appears the assumption is that the butter is adulterated. Evidently this inference can no longer be regarded as accurate. It does not follow that the Wolny test should be abandoned, but that it should be differently applied. If by law it was demanded that commercial "butter" must needs show the fixed proportion of volatile fatty acids, without reference to whether or not it claimed to be made entirely from milk, a standard would be fixed which at present, it would seem, is non-existent. Butter made from inferior milk would rank only as margarine, in that it lacked the special characteristics of good butter. It is true that there is no difference in the nutritive qualities of margarine and butter. But there is a difference in flavour and in price, and if bad butter were not sold at the price of good there would be a chance of the standard being raised.

